# hsa_circ_0000518 Facilitates Non-Small-Cell Lung Cancer Progression via Moderating miR-330-3p and Positively Regulating SLC1A5

**DOI:** 10.1155/2022/4996980

**Published:** 2022-07-15

**Authors:** Huilai Lv, Zhihua Shi, Aixia Sui, Yan Zhang, Liangbiao Peng, Mingbo Wang, Fan Zhang

**Affiliations:** ^1^Department of Thoracic Surgery, The Fourth Hospital of Hebei Medical University, Shijiazhuang, Hebei Province 050000, China; ^2^Department of Oncology, Hebei General Hospital, Shijiazhuang, Hebei Province 050000, China; ^3^Department of Oncology, Shijiazhuang People's Hospital, Shijiazhuang, Hebei Province 050000, China; ^4^Department of Thoracic Surgery, The Fourth Hospital of Handan, Handan, Hebei Province 056000, China

## Abstract

**Background/Aim:**

Non-small-cell lung cancer (NSCLC) is the principal agent of cancer deaths globally. The goal of this study was to determine how circular RNA_0000518 (circ_0000518) regulates tumor progression. *Materials/Methods*. circ_0000518 was selected as a study target involved in NSCLC from GEO (Gene Expression Omnibus) database. circ_0000518 level was gauged by qRT-PCR. It was confirmed as circRNA by actinomycin D inhibition and RNase R assay. Subcellular localization of circ_0000518 was identified by FISH. Cell function was determined by CCK-8, Transwell, and western blot. Glutamine metabolic factors were detected by ELISA. The target regulation relationship between genes was clarified by dual-luciferase reporter assay. In vivo models were established to evaluate the impact of circ_0000518 on tumor growth. Immunohistochemical staining for Ki67, vimentin, and E-cadherin was used to detect cell proliferation and metastasis, respectively.

**Results:**

circ_0000518 expression was enhanced in NSCLC. si-circ_0000518 inhibited cell proliferation, invasion, and glutamine metabolism. circ_0000518 functioned as a molecular sponge for miR-330-3p, and inhibition of miR-330-3p in cells markedly reversed circ_0000518 interference-mediated antitumor effects. miR-330-3p interacted with 3′-UTR of SLC1A5. miR-330-3p inhibitor-mediated protumor effect was remarkably reversed in cells after the knockdown of SLC1A5. circ_0000518 knockdown reduced glutamine, glutamate, and *α*-KG by targeting miR-330-3p. Intertumoral injection of circ_0000518 shRNA adeno-associated virus effectively halted xenograft tumor growth.

**Conclusion:**

The current study revealed that circ_0000518 may have a prooncogenic function in the formation and progression of NSCLC, which might be achieved through moderating the miR-330-3p/SLC1A5 axis.

## 1. Introduction

Lung cancer has been one of the major challenges in antineoplastic therapy. Lung cancer accounts for 11.4% of the incidence and 18% of the mortality of all tumors. Among them, non-small-cell lung cancer (NSCLC) is its main pathological type, accounting for over 80 percent of all primary lung cancer cases [1]. Early diagnosis can enhance the prognosis of people with lung cancer. However, most individuals diagnosed are at an advanced stage, leading to ineffective therapy [2]. High recurrence rates and susceptibility to distant metastasis are the leading causes of increased mortality in lung cancer [3]. Therefore, elucidating the underlying molecular mechanisms in NSCLC development is critical for NSCLC sufferers' therapeutic care.

Circular RNAs (circRNAs) are extensively distributed in various human tissues and perform a critical part in tissue remodeling angiogenesis, embryogenesis, and tumor development [4–6]. circRNA can lift the repressive effect of miRNAs on their target genes through miRNA sponge action [7, 8]. circRNAs are irregularly expressed in countless tumors and are involved in regulating NF-*κ*B, PI3K/AKT, TGF-*β*, and JAK-STAT signaling pathways [9–12], thereby affecting tumor development. It was shown that circVANGL1 in NSCLC inhibits miR-195 by adsorbing miR-195 and thus negatively regulates downstream genes [13]. circBIRC6 expressed in NSCLC can similarly inhibit miR-145 involved in NSCLC progression through sponge effect [14]. circ_0000518 is a circular RNA of 150 bp in length consisting of reverse splicing of the Ribonuclease P RNA Component H1 (RPPH1) gene. Preliminary research indicated that circ_0000518 promotes breast cancer development [15]. Therefore, it is necessary to investigate the part of circ_0000518 that evolved in NSCLC.

Glutamine is considerable noncritical amino acid within human body. In contrast, glutamine metabolism is greatly elevated in cells with high proliferation capacity, including inflammatory cells, stem cells, and lung cancer cells [16–18]. Glutamine must be transported through a specific carrier on the cell membrane to enter the cell for its action [19]. Among them, the Na^+^-dependent glutamine carrier named solute carrier family 1 member 5 (SLC1A5) is pivotal [20]. SLC1A5 is widely expressed in normal tissues. In addition, SLC1A5 is significantly increased in various neoplasms [21, 22], indicating that SLC1A5 is closely related to human physiological functions and many major diseases.

In this study, circ_0000518 expression was markedly elevated in NSCLC. Circ_0000518 knockdown quenched cell proliferation, invasion, and glutamine metabolism. Mechanistically, circ_0000518 knockdown exerted its oncogenic effect by adsorbing miR-330-3p to regulate SLC1A5. To confirm the effects of circ_0000518 within the progression of NSCLC, this study used mouse transplantation tumor experiment to observe the influence of circ_0000518 on NSCLC growth, revealing the part of circRNAs.

## 2. Materials and Methods

### 2.1. Subjects and Samples

160 patients with confirmed NSCLC from April 2019 to August 2021 were collected from the Fourth Hospital of Hebei Medical University. There were 98 males and 62 females, with a mean age of 62.62 ± 9.61 years and 56.1 ± 9.87 years, respectively. Clinical information of NSCLC patients included gender, age, smoking history, lymph node metastasis, and clinical stage, excluding patients who received previous neoadjuvant chemotherapy or history of malignant tumors, and seeking patients' consent and signing the informed consent form. Patients were also asked for their consent and signed an agreement of acknowledgment. General facts for the cases are shown in Table 1.

### 2.2. Database Setup

We searched GEO (http://www.pubmed.com/geo) to identify dataset suitable for the analysis. In this, the keywords “NSCLC”, “survival”, and “GPL19978” were used. The GSE101586 matrix data file was downloaded from the GEO database, containing data from 5 NSCLC tissue samples (experimental group) and 5 normal lung tissue samples (control group). The differential circRNA screening criteria were *p* < 0.01 and differential fold change ∣logFC | ≥1.5.

### 2.3. Cell Culture

NSCLC A549 and H1299 cells, normal lung bronchial epithelial cells, and embryonic kidney cells 293T were purchased from CellBank (Shanghai, China). A549 and 293T cells were cultured in DMEM medium containing 10% FBS, H1299 cells were grown in RPMI 1640 medium containing 10% FBS, and BEAS-2B cells were grown in DMEM/F12 culture medium. All cells were incubated in a sterile constant temperature incubator at 37°C with 5% CO_2_. The cells were passaged after they reached an 80%-90% fusion level. Experiments were performed during the logarithmic growth period of cells.

### 2.4. Quantitative Real-Time PCR (qRT-PCR)

Total RNA was extracted from each group of cells using TRIzol reagent and tested for concentration, then reverse transcribed into cDNA depending on the operating directions of TaKaRa (Dalian, China), and amplified for detection. Primers were synthesized by Pharma (Suzhou, China), and Table 2 displays the primer sequences. The qRT-PCR reaction system was 20 *μ*L, including 20× SYBR Green 0.3 *μ*L, 10× PCR buffer 2 *μ*L, 10 *μ*mol/L forward and reverse primers 0.5 *μ*L each, 10 mmol/L dNTPs 0.5 *μ*L, 1 *μ*L reverse transcription product, and 5 U/*μ*L DNA polymerase 0.2 *μ*L. The reaction conditions were predenaturation 95°C for 60 s, 95°C for 5 s, and 60°C for 40 s. The amplification efficiency of PCR was judged by the amplification curve, and the specificity of PCR products was detected by the lysis curve. Relative expression of circ_0000518, miR-330-3p, and SLC1A5 mRNAs was measured by beta-actin gene-level correction using 2^-*ΔΔ*Ct^ analysis.

### 2.5. RNase R Digestion Assay

The extracted RNA was divided into RNase R-treated and control groups, each with 10 *μ*g. The RNase R-treated group was treated with 20 U RNase R (2 U/*μ*g), and the control group was incubated with an equal amount of double-distilled water for 10 min at 37°C. Equal amounts of digestion products were taken from both groups and reverse transcribed into cDNA. circ_0000518 and RPPH1 mRNA expression were detected. *β*-Actin was chosen as the internal referential gene.

### 2.6. Actinomycin D Assay

Resuscitation A549 and H1299 cell lines were cultured for one generation. Cells were counted and divided equally into 5 portions and inoculated into 5 wells of a 6-well plate at 0.8 × 10^5^ cells. When the cell growth density reached 80%, the cells in the wells were collected, and the RNA was extracted, while actinomycin D was added to the remaining wells to an ultimate concentration of 2 *μ*g/mL. After 4, 8, and 12 h, respectively, the cells in wells 2-5 were collected, and the RNA was extracted, followed by qRT-PCR.

### 2.7. Fluorescence In Situ Hybridization (FISH)

Cells were incubated with circ_0000518 probe with enclosures at 37°C overnight. Cells were stained with DAPI, and the DS-U3 (Nikon, Japan) microscope was fluorescence imaging.

### 2.8. Cell Transfection

Small interfering RNA targeting circ_0000518 (si-circ_0000518) or SLC1A5 (si-SLC1A5) was constructed. GenePharma (Suzhou, China) manufactured their control (si-NC), short hairpin RNA targeting circ_0000518 (sh-circ_0000518) and its control (sh-NC), miR-330-3p mimic and control mimics (miRNA NC), miR-330-3p inhibitor, and inhibitor NC. Cell transfection was conducted employing Lipofectamine 2000 (Invitrogen).

### 2.9. Cell Counting Kit-8 (CCK-8) Assays

NSCLC cells at the logarithmic phase were harvested and resuspended with corresponding culture medium, and the CCK-8 reagent was added separately. The cells were incubated in an incubator at 37°C and CO_2_ volume fraction of 5% for 2 h. Then, they were put into the enzyme marker for detection. The remaining wells were incubated for 24, 48, and 72 h, respectively, with the addition of 20 *μ*L CCK-8 reagent. Then, optical density (450 nm) was detected using an enzyme marker.

### 2.10. Transwell Invasion Assay

NSCLC cells were suspended in serum-free medium and inoculated in the upper layer of Transwell chambers. After incubation in 5% CO_2_ 37°C for 24 h, the upper layer of culture medium was aspirated and carefully wiped off to remove any cells that had not yet invaded the chambers. Subsequently, the chambers were placed into 1% crystal violet staining for 30 min and then counted under a microscope.

### 2.11. Western Blot

Western blot analysis was conducted as previously described [23]. Cells were lysed in RIPA protein lysis solution. Equal amounts of proteins were separated by SDS-PAGE and then transferred onto PVDF membranes. The membranes were washed and then incubated with primary antibodies (anti-Ki-67 (ab92742, Abcam, 1 : 1000), anti-E-cadherin (ab1416, 1 : 500), antivimentin (ab8979, 1 : 1000), anti-*β*-actin (ab8226, 1 : 1000), anti-SLC1A5 (ab237704, 1 : 1600)) overnight at 4°C, followed by incubation with horseradish peroxide- (HRP-) labeled secondary antibody at 37°C for 2 h. Band intensity was quantified by the ImageJ software.

### 2.12. Enzyme-Linked Immunosorbent Assay (ELISA)

Glutamate measurements were determined by ELISA as described previously [24]. The human glutamine (Gln) ELISA kit, human glutamate (Glu) ELISA kit, and human *α*-ketoglutaric acid (*α*-KG) ELISA kit instructions were followed to detect Glu, Gln, and *α*-KG levels in NSCLC cells and tumors. The results were calculated as the corresponding control levels 1 and the levels of glutamate and *α*-KG in the cell supernatant of the assay group.

### 2.13. Bioinformatic Analysis

The target miRNAs of hsa-circ_0000518 were predicted using CircInteractome (https://circinteractome.nia.nih.gov) and ENCORI online prediction website (https://starbase.sysu.edu.cn), and the predictions were intersected to obtain the target miRNAs of hsa-circ_0000518. The ENCORI database was used to predict the target genes of miR-330-3p.

### 2.14. RNA Immunoprecipitation (RIP) Assay

The binding of hsa_circ_0000518 to Ago2 protein was detected by RIP kit (MAGNARIP02, Millipore, USA). Cell extracts were incubated with antibodies for coprecipitation, and magnetic beads were washed and resuspended with 5 *μ*g of antibody Ago2 (ab186733, 1 : 40) and IgG (ab205718, 1 : 500) incubated. Magnetic bead-antibody complexes were washed, resuspended, and incubated overnight at 4°C by adding 100 *μ*L cell extracts. Proteinase K digestion was used to derive RNA from the samples.

### 2.15. Dual-Luciferase Reporter Assay

Synthesize hsa_circ_0000518 fragment containing specific miR-330-3p binding site or mutant portion and insert into pmirGLO vector (Promega, Madison, WI, USA). 3′-UTR of SLC1A5 with a specific miR-330-3p binding site or mutant sequence was constructed and cloned into the pmirGLO vector. Cells were cotransfected with miR-330-3p mimic or miRNA NC in these plasmids.

### 2.16. Mouse Xenograft Assay

Transplant tumor models were performed as described previously [25]. All animal experiments were accredited by the Hospital Animal Care and Use Committee of the Fourth Hospital of Hebei Medical University. Female thymus-free BALB/c nude mice (4 weeks old) were obtained from Vitalriver (Beijing, China). Mice were housed in a pathogen-free environment (24 ± 2°C, 55 ± 5% humidity) with a 12 h light-12 h dark cycle; during the whole experimental period, food and water were supplied ad labitum. The mice were assigned into sh-NC/sh-circ_0000518 groups at random, with 5 mice in each group. A549 cells (1 × 10^7^ cells) with knockdown circ_0000518 (sh-circ_0000518) and negative control cells were injected subcutaneously into the right abdomen of mice, and tumor volume and mass were monitored every 7 days. Euthanasia was performed through CO_2_ asphyxiation in step with protocol on day 28 after injection as described before [26], and xenograft tumors were excised and weighed.

### 2.17. Immunohistochemistry (IHC)

Paraffin sections made from tumor tissue were prebaked at 80°C for 2 h. After dewaxing, 3% H_2_O_2_ solution was employed to block peroxidase for 10 min. Then, the slices were microwave heated in sodium citrate buffer for antigen repair. After serum closure for 30 min, the section cutting was incubated with first antibodies (anti-Ki-67 (ab92742, Abcam, 1 : 1000), anti-E-cadherin (ab1416, 1 : 500), antivimentin (ab8979, 1 : 1000)) overnight at 4°C. HRP-labeled polymer-coupled secondary antibody stained the sections for 1 h. The immune complexes were stained with DAB for 5 min, and nuclei were restained with hematoxylin for 30 s. The sealed slices were allowed to dry and then observed under a microscope for photographs.

### 2.18. Statistical Analysis

All data were processed with the SPSS 21.0 statistical software (SPSS Inc., USA) and represented as mean ± SD. A paired *t*-test was performed to assess cancerous versus paracancerous tissues. One-way analysis of variance (ANOVA) followed by LSD post hoc tests was used to compare multiple groups. Pearson's analysis was utilized to assess the expression and correlation of hsa_circ_0000518 and miR-330-3p. All experiments were replicated thrice.

## 3. Results

### 3.1. circ_0000518 Is Highly Expressed in NSCLC

**Figure 1:**
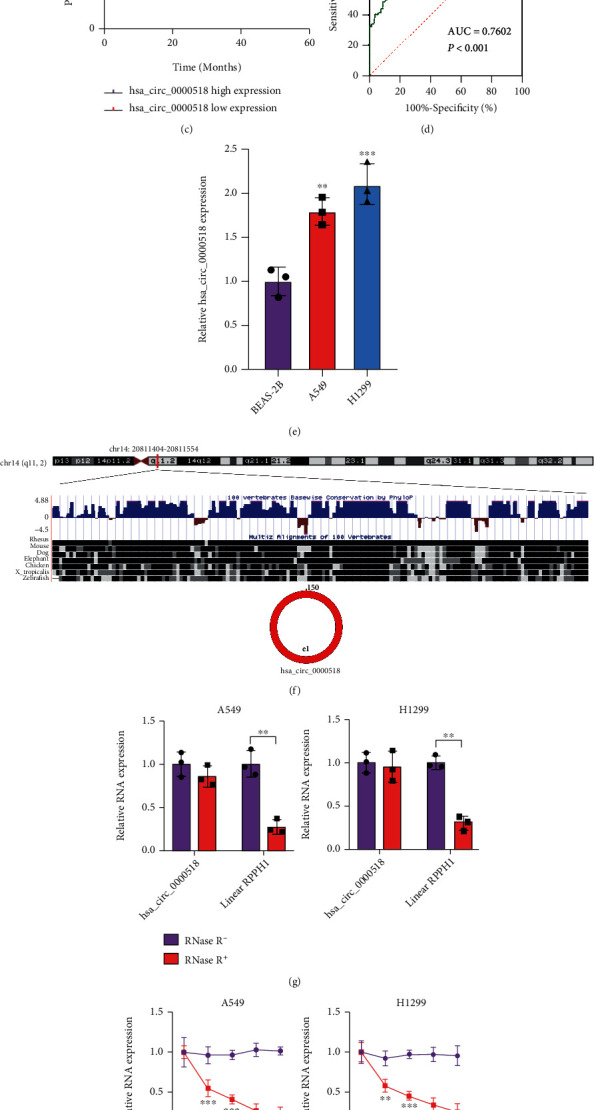
circ_0000518 is relatively highly expressed in NSCLC tissues and cell lines and predominantly localized in the cytoplasm. (a) Gene Expression Omnibus (GEO) dataset analysis of the different expressions of circ_0000518 between NSCLC and normal tissues. (b) The expression of circ_0000518 was detected by qRT-PCR in NSCLC and adjacent noncancerous tissues. (c) Kaplan-Meier study of the association between circ_0000518 expression and overall survival in patients with NSCLC. (d) The ROC curve was used to assess the possible diagnostic value of circ_0000518; the AUC was 0.7602. (e) circ_0000518 expression in NSCLC cells was detected by qRT-PCR. (f) Chromosomal locations of circ_0000518. (g) qRT-PCR analysis of circ_0000518 and RPPH1 expression levels after treatment with RRPPH1 RNase R. (h) qRT-PCR analysis for the half-life of circ_0000518 after treatment with actinomycin D. **(**i) FISH analysis for the localization of circ_0000518. ^∗∗^*p* < 0.01, ^∗∗∗^*p* < 0.001.

The GSE101586 dataset was downloaded from Gene Expression Omnibus (GEO) and subsequently analyzed for differential genes by GEO2R. circ_0000518 was notably expressed in NSCLC tissue samples (Figure 1(a)). circ_0000518 expression was greater in NSCLC than in paraneoplastic tissues (Figure 1(b)). High circ_0000518 expression was linked to bleak prognosis in NSCLC sufferers (Figure 1(c)). ROC analysis was utilized to quest the diagnostic accuracy of circ_0000518 for separating patients at the clinical stage I from healthy controls, to further explore its potential for early identification of NSCLC. The AUC value was 0.760, which had some diagnostic value, according to the data (Figure 1(d)). By analyzing the relationship between circ_0000518 expression and clinicopathological parameters, high circ_0000518 expression was linked to tumor size, TNM stage, tumor infiltration depth, and lymph node metastasis (Table 1). Besides, circ_0000518 expression was remarkably greater in both NSCLC cells (Figure 1(e)). The position and structure of circ_0000518 on the chromosome are shown in Figure 1(f). RNase R treatment reduced the linear transcript RPPH1 mRNA level corresponding to circ_0000518, and the expression level of the cyclic transcript of circ_0000518 was not significantly changed (Figure 1(g)). Actinomycin D could inhibit intracellular RNA transcription, and circ_0000518 had better RNA stability than linear RPPH1 mRNA after 24 h treatment by actinomycin D (Figure 1(h)). circ_0000518 was predominantly localized in the cytoplasm (Figure 1(i)). The results suggest that high expression of circ_0000518 may be associated with NSCLC advancement.

### 3.2. si-circ_0000518 Restrains Cell Malignancy

**Figure 2:**
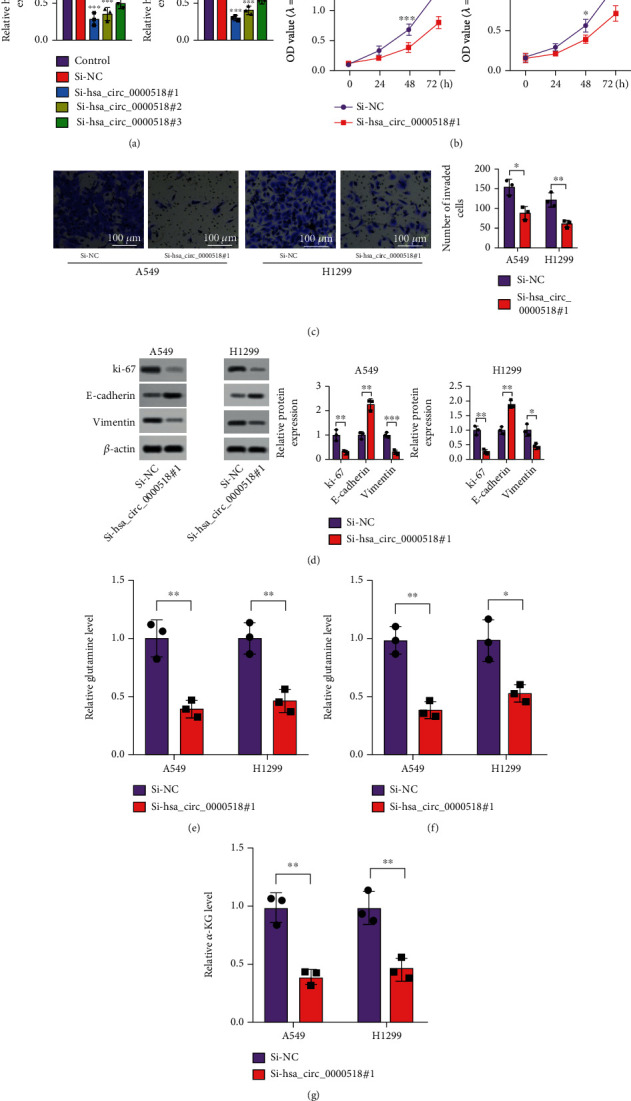
circ_0000518 knockdown restrains NSCLC cell proliferation, invasion, and glutamine metabolism. (a) Relative circ_0000518 levels in cells after the si-circ_0000518 knockdown were evaluated by qRT-PCR. (b) The effects of si-circ_0000518 on NSCLC cell viability were detected by the CCK-8 assay. (c) circ_0000518 knockdown inhibited NSCLC cell invasion by Transwell assay. (d) Effects of knockdown of circ_0000518 on vimentin, E-cadherin, and Ki-67 expression. (e–g) Glutamine, glutamate, and *α*-KG levels were examined. ^∗^*p* < 0.05, ^∗∗^*p* < 0.01, ^∗∗∗^*p* < 0.001.

Then, we transfected cells with si-circ_0000518 for the construction of a cell model with low circ_0000518 expression (Figure 2(a)). Subsequent knockdown of circ_0000518 remarkably quenched cell proliferation (Figure 2(b)). Cell invasion assay indicated that si-circ_0000518 lessened cell invaded number (Figure 2(c)). Further detection of cell proliferation and EMT-related protein expression evinced that si-circ_0000518 constrained Ki-67 and vimentin and promoted E-cadherin protein expression (Figure 2(d)). Finally, we examined the levels of glutamine metabolism-related factors; as a result, the content of glutamate, glutamine, and *α*-KG was reduced by knockdown of circ_0000518 (Figure 2(e)). Silencing circ_0000518 effectively inhibited NSCLC cells' capacity to grow and invade, along with glutamine metabolism.

### 3.3. circ_0000518 Interacts with miR-330-3p

**Figure 3:**
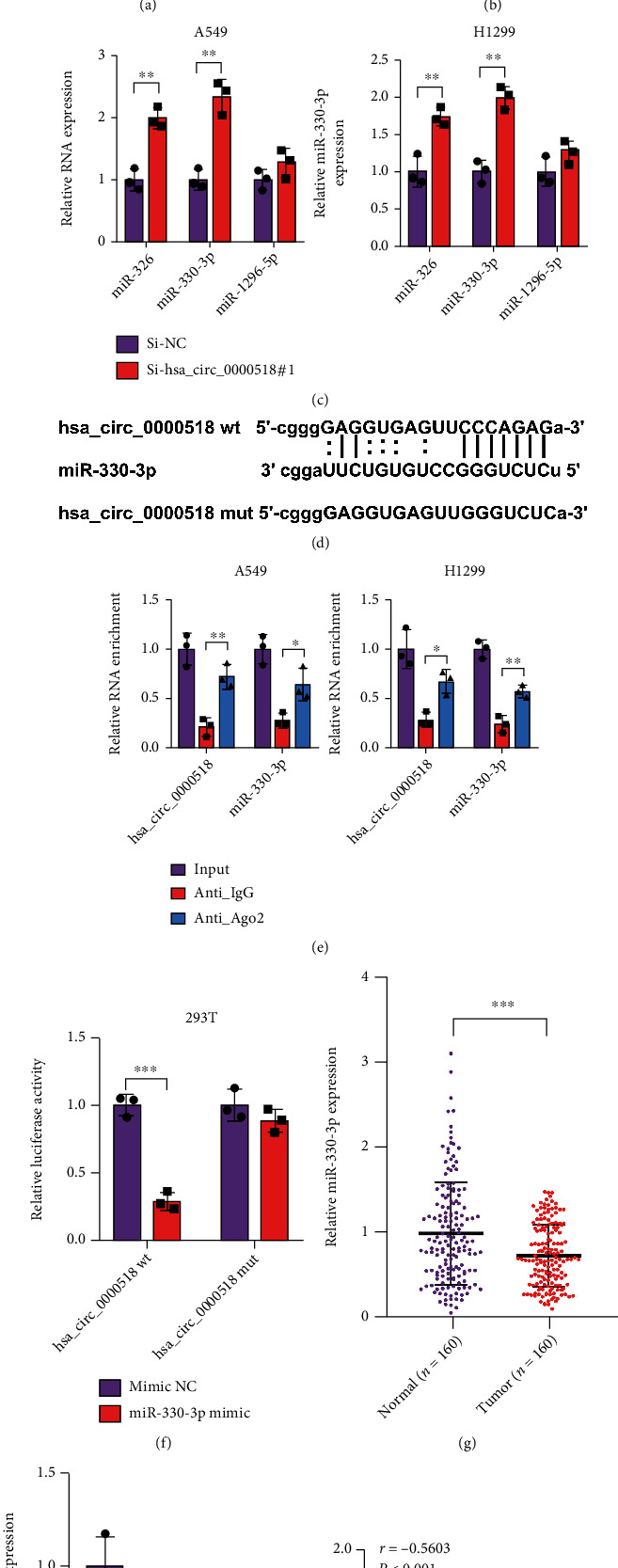
circ_0000518 had a negative correlation with miR-330-3p. (a) miRNAs were computationally predicted using CircInteractome and ENCORI databases. (b) Differences in miRNA expression in tumor and normal tissues. (c) Detections of the targeted microRNAs (miR-326, miR-330-3p, and miR-1296-5p) in different cancer cell lines. (d) The binding sites of miR-330-3p with wt or mut circ_0000518 3′UTR are shown schematically. (e) Enrichment levels of circ_0000518 and miR-330-3p extracted from Ago2 protein were analyzed by RIP. (f) Luciferase reporter assay of 293T cells cotransfected with circ_0000518-wt or circ_0000518-mut and miR-330-3p or the miR-330-3p-NC. (g) miR-330-3p expression levels in NSCLC vs. paired adjacent nontumor tissue (*n* = 160). (h) The expression of miR-330-3p in NSCLC and normal cell line was measured by qRT-PCR. (i) circ_0000518 expression was negatively correlated with miR-330-3p expression. ^∗^*p* < 0.05, ^∗∗^*p* < 0.01, ^∗∗∗^*p* < 0.001.

Bioinformatics methods were utilized to ascertain relevant downstream targets of circ_0000518 for investigating the probable mechanism of circ_0000518 in NSCLC. The predicted regulators for circ_0000518 comprised miR-326, miR-330-3p, and miR-1296-5p (Figure 3(a)). All of the above miRNAs were underexpressed within NSCLC, while miR-330-3p was the most significant (Figure 3(b)). We observed that si-circ_0000518 could remarkably increase miR-330-3p expression after downregulating circ_0000518 at the ex vivo level (Figure 3(c)). The binding affinity was confirmed between miR-330-3p and circ_0000518. The antibodies against Ago2 could precipitate circ_0000518, thus competitively binding miR-330-3p (Figure 3(e)). To further demonstrate that circ_0000518 can directly target miR-330-3p, we constructed fluorescent reporter gene plasmid vectors comprising the miR-330-3p binding target (wt-circ_0000518) and mutation site (mut-circ_0000518). After cotransfection of the two fluorescent vectors into 293T cells, miR-330-3p greatly impaired the luciferase activity within transfected circ_0000518 cells (Figure 3(f)). miR-330-3p was markedly hypoexpressed among NSCLLC clinical samples (Figure 3(g)), and in NSCLC cell lines, the same phenomenon existed (Figure 3(h)). Furthermore, circ_0000518 and miR-330-3p were shown to have an adverse correlation (Figure 3(i)). Therefore, the results suggest that circ_0000518 directly targets miR-330-3p.

### 3.4. miR-330-3p Inhibitor Partially Reversed the Inhibitory Effect of si-circ_0000518 on Cell Malignancy

**Figure 4:**
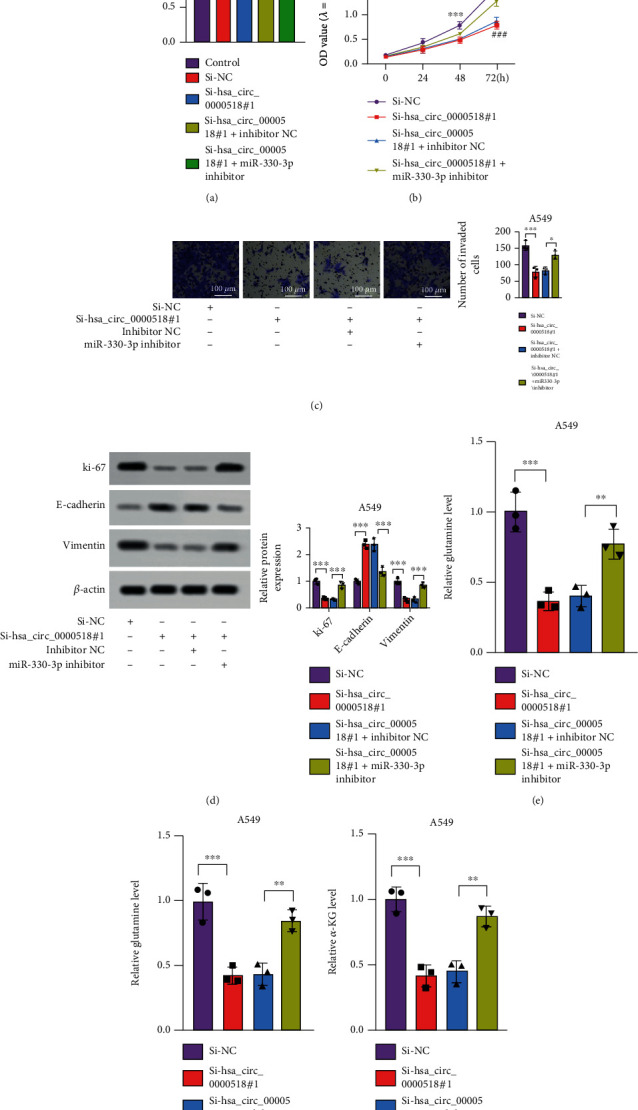
Downregulation of miR-330-3p contributes to inhibition of proliferation, invasion, and glutamine metabolism of NSCLC cells. (a) Transfection efficiency of miR-330-3p inhibitor determined using qRT-PCR. (b) CCK-8 assays were performed in cells stably transfected with NC or si-circ_0000518, with or without the miR-330-3p inhibitor. (c) Transwell invasion assays with A549 cells stably transfected with the NC vector or si-circ_0000518, with or without the miR-330-3p inhibitor. (d–g) Ki-67, E-cadherin, and vimentin levels in A549 cells. ^∗∗^*p* < 0.01, ^∗∗∗^*p* < 0.001.

si-circ_0000518 induced miR-330-3p expression; however, the miR-330-3p inhibitor restored it (Figure 4(a)). The biological behavior of NSCLC cells regulated by circ_0000518 thru miR-330-3p was investigated and concluded that si-circ_0000518 transfection had an inhibitory effect on the proliferation, invasion, and EMT of NSCLC cells, but this effect was partially rescued by miR-330-3p inhibitor (Figures 4(b)–4(g)). These findings imply that circ_0000518 promotes NSCLC cell proliferation, invasion, and glutamine metabolism through competitive binding to miR-330-3p.

### 3.5. miR-330-3p Binds to SLC1A5-3′UTR

**Figure 5:**
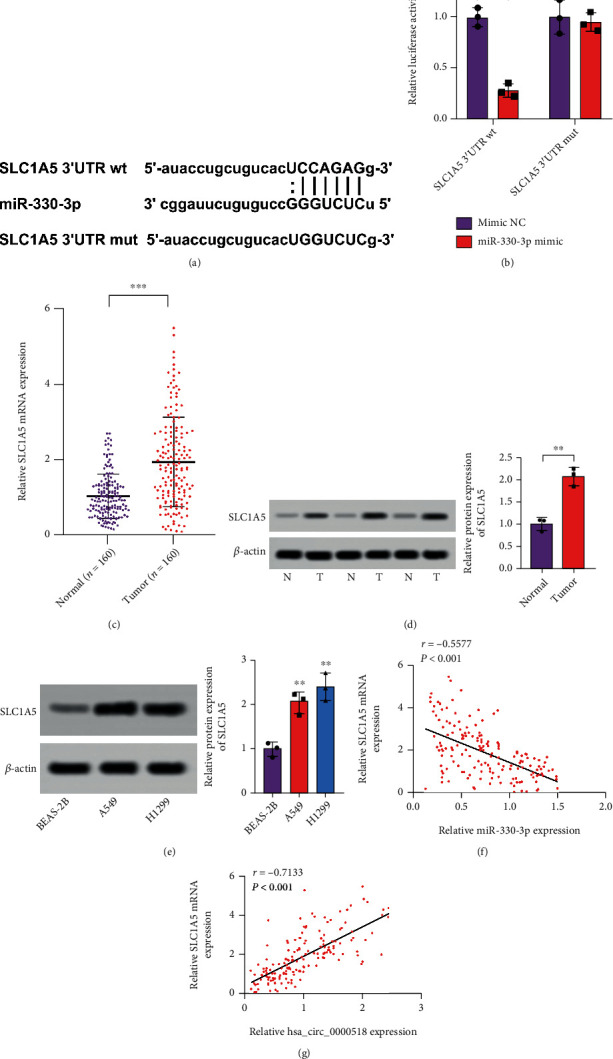
SLC1A5 is a direct regulator to miR-330-3p. (a) The putative binding sites of miR-330-3p on SLC1A5. (b) Dual-luciferase assays confirmed the interaction of SLC1A5 with miR-330-3p. (c) The transcriptional level of SLC1A5 mRNA in NSCLC and normal tissue pairs (*n* = 160) was determined by qRT-PCR. (d) SLC1A5 protein expression was assessed by western blot in 3 paired NSCLC and normal tissues. (e) The level of SLC1A5 protein expression was assessed by western blot in normal and NSCLC cells. (f) miR-330-3p and SLC1A5 levels are inversely related. (g) Positive correlation between hsa_circ_0000518 and SLC1A5. ^∗∗^*p* < 0.01, ^∗∗∗^*p* < 0.001.

We next predicted and discovered that SLC1A5 and miR-330-3p existed in base complementary sequences by bioinformatics software RNA22. The binding sites of miR-330-3p to SLC1A5 and sequences of mutant SLC1A5 are shown in Figure 5(a). miR-330-3p could target SLC1A5-*3*′*UTR* (Figure 5(b)). SLC1A5 expression was shown to be greater in NSCLC than in ordinary tissues, and similar findings were obtained at cellular levels (Figures 5(c)–5(e)). The expression of SLC1A5 mRNA and miR-330-3p had an adverse connection (Figure 5(f)) but positively correlated with circ_0000518 expression (Figure 5(g)). SLC1A5 was a downstream regulator for miR-330-3p; then, circ_0000518 adsorbed miR-330-3p before regulating SLC1A5 expression.

### 3.6. sh-circ_0000518 Restricted Tumor Growth In Vivo

**Figure 6:**
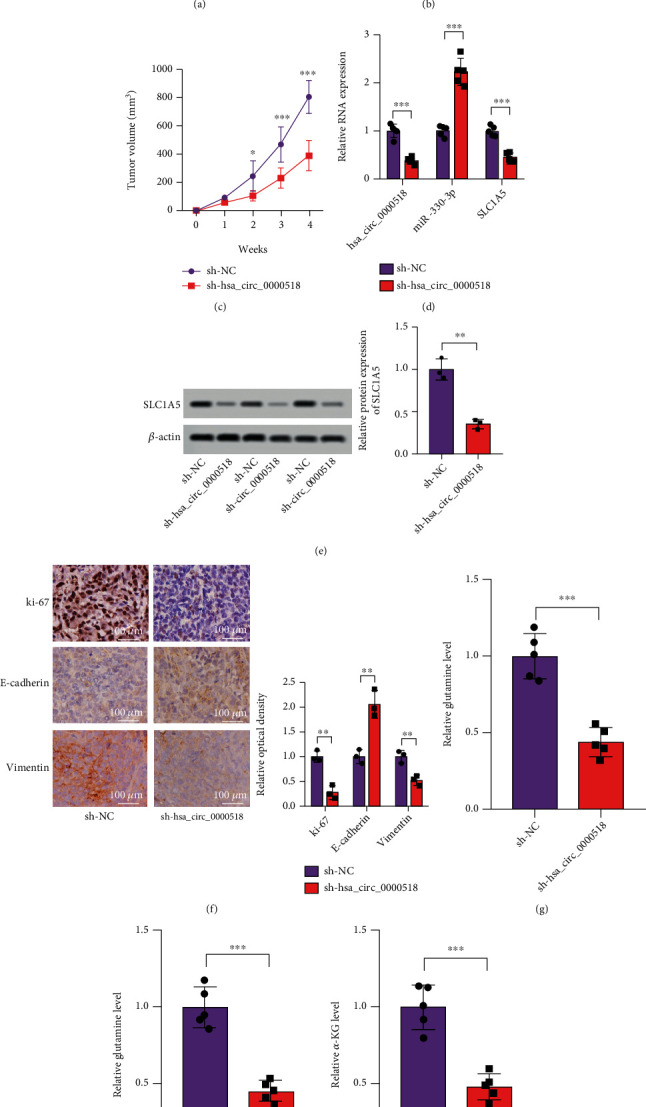
sh-circ_0000518 restrains tumor development. (a) The nude mice's weight from each group did not change significantly during the experiment. (b) Tumor weights were plotted and showed that circ_0000518 silencing has lower tumor weights. (c) Tumor volume curve was analyzed. (d, e) circ_0000518, miR-330-3p, and SLC1A5 levels within xenograft tumors by qRT-PCR and western blot assays. (f) Immunohistochemistry staining for Ki-67, E-cadherin, and vimentin of the tumor sections in both the groups (×400). (g–i) Levels of glutamine, glutamate, and *α*-KG in the sh-circ_0000518 groups and sh-NC groups were measured by ELISA. The photographs are representative. ^∗^*p* < 0.05, ^∗∗^*p* < 0.01, ^∗∗∗^*p* < 0.001.

There is further *in vivo* validation of circ_0000518 against NSCLC. Mice were injected with A549 cells transfected with sh-NC/sh-circ_0000518. Tumor volume and weight were found to be lower in the sh-circ_0000518 group than in the sh-NC group (Figures 6(a)–6(c)). The knockdown of circ_0000518 led to a considerable diminishment in SLC1A5 mRNA expression (Figure 6(d)). Following that, SLC1A5 protein within the sh-circ_0000518 group was depressed compared to the normal control shRNA group (Figure 6(e)). IHC staining of isolated tumor tissues showed that sh-circ_0000518 decreased the protein expression of Ki-67 and vimentin with elevated levels of E-cadherin **(**Figure 6(f)). Consistent with the cellular level, sh-circ_0000518 reduced the glutamine metabolic factors Glu, Gln, and *α*-KG content (Figures 6(g)–6(i)). circ_0000518 knockdown can inhibit tumorigenic ability in nude mice *in vivo*, according to experimental findings.

## 4. Discussion

circRNAs are expected to have a critical role in the advancement of NSCLC. For instance, TIF1*γ* is controlled by circPTK2, which prevents the epithelial-mesenchymal transition of NSCLC cells [27]. circ_0067934 controls lung cancer cell proliferation via Wnt/*β*-catenin [28]. We unearthed that circ_0000518 expression was enhanced, and excessive circ_0000518 expression was related to poor prognosis in NSCLC patients. The correlation analysis between the expression level of circ_0000518 and clinicopathological characteristics in the case subgroups showed that the expression level of circ_0000518 correlated with tumor size, TNM stage, depth of invasion, lymph node metastasis, and distant metastasis degree, indicating that circ_0000518 promoted the growth and metastasis of NSCLC cells. The lower the degree of tumor differentiation the faster the growth, the higher the malignancy, and the poor prognosis of patients. The high expression of circ_0000518 has some theoretical value for the diagnosis and prognosis assessment of NSCLC.

Treatment with RNase R and actinomycin D confirmed that circ_0000518 is a circular transcript. The biologic activity of circ_0000518 was next outlined using knockdown techniques. Glutamine metabolism has been implicated in both the growth and survival of cancer cells, and high glutamine levels contribute to tumor development. Glutamine catabolism produces *α*-KG, which supports cancer cell proliferation and tumor growth [29]. In the current study, circ_0000518 knockdown quenched cell proliferation, invasion, and degree of glutamine metabolism. These illustrated that silencing circ_0000518 effectively slowed the development of NSCLC.

Notably, circRNAs can behave like sponges and thus regulate downstream gene expression by acting as endogenous competitive RNAs. For example, by functioning as a sponge for miR-328-5p, cirCAMSAP1 suppresses the advancement of colorectal cancer [30]. circSETD3 acts as ceRNA to competitively binding miR-421 to suppress hepatocellular carcinoma growth [31]. In addition, circPRMT5 acts as a promoter of gastric carcinoma progression by inhibiting miR-145 and miR-1304 [32]. Research implied that circ_0000518 moderates miR-326 to accelerate breast cancer advancement [15]. Given that circ_0000518 exerts opposite effects to miR-330-3p within NSCLC, we speculate that those two have a comparable targeting relation as indicated above. Interestingly, there are binding sites unearthed from circ_0000518 to miR-330-3p utilizing bioinformatics research. The ability of circ_0000518 to sponge miR-330-3p was validated. Additionally, knockdown of circ_0000518 caused increased miR-330-3p expression. On other side, the inhibitory impacts of circ_0000518 silencing with cells were incompletely reversed by inhibiting miR-330-3p. As a result of the adsorption of them by circ_0000518, we determined that it was engaged in controlling NSLCSC cell proliferation, invasion, and glutamine metabolism.

Since miRNAs are implicated in the modulation of genetic expression, further research into their function in cancer advancement is required. Several anterior types of research have unearthed that miR-330-3p serves as one suppressor in a wide diversity of cancers. It is negatively controlled by circ_0016068 and then regulates BMI-1 to suppress prostate cancer cell growth [33]. miR-330-3p as lncTPT1-AS1 downstream and regulates QKI and thus inhibits the malignancy of breast cancer cells [34]. A parallel situation existed in respect of miR-330-3p within NSCLC. It restrained tumor advancement through regulating EGR2 in NSCLC [35]. We propose with miR-330-3p being downregulated within NSCLC and controlled by circ_0000518 and its inhibition reversed the contribution of silencing circ_0000518 to tumor growth. miR-330-3p's biological function could be attributed to targeting SLC1A5, thereby achieving restraint on cell malignancy.

Glutamine is the predominantly plentiful essential nutrient and an important metabolic precursor in the human body, contributing to nucleotide and protein as well as glutathione synthesis [36]. It has been found that glutamine promotes tumor proliferation, invasion, and stimulating cardiovascular production in tumor tissues [37–39]. The role of SLC1A5 as the most important transporter of glutamine in tumors has naturally attracted much attention. Studies have shown that restricting glutamine uptake by blocking SLC1A5 can cause MYC-dependent apoptosis [40]. SLC1A5 is aberrantly highly expressed in breast cancer [41]. Studies in neuroblastoma cell lines also found high expression of SLC1A5 and confirmed that Na^+^-dependent glutamine uptake accounted for more than 95% of the total glutamine uptake in this cell [42]. In addition, tumors with high SLC1A5 expression have been found in recent years, including prostate and lung cancer [43, 44]. Previous studies have shown that circSFMBT2 drove aggressive esophageal carcinoma phenotypes through the miR-107 dependence modulation for SLC1A5 [45]. Moreover, circ_0000463 is dedicated to the development and glutamine metabolism of NSCLC by targeting miR-924/SLC1A5 signaling [46], which is consistent with our work. SLC1A5 was considerably elevated and controlled by miR-330-3p negatively. Glu, Gln, and *α*-KG levels were also significantly lower in tumors with knockdown of SLC1A5. Knockdown of SLC1A5 restored the inhibition of NSCLC cell malignancy by silencing circ_0000518.

Taken together, this study suggested that circ_0000518 was upregulated and might be associated with deteriorated prognosis in NSCLC. Therefore, it could function as a latent biomarker and prognostic indicator for NSCLC. circ_0000518 knockdown arrested the proliferation, invasion, and glutamine metabolism by targeting miR-330-3p/SLC1A5, thus shedding new light on the mechanisms involved in the development of NSCLC. However, this study also has certain limitations. We tested the effects of circ_0000518 knockdown alone but not the influence of circ_0000518 overexpression on NSCLC. Furthermore, there is a lack of corroboration of the signaling pathway and more experimental validation of the cellular phenotype. Therefore, further experiments are necessary to offer more profound evidence for continued studies.

## Figures and Tables

**Table 1:** Correlations between hsa_circ_0000518 levels and clinicopathological features of patients.

Characteristics	Cases	hsa_circ_0000518		*p* value
		Low	High	
Age (years)				0.5153
≤60	68	38	30	
>60	92	57	35	
Gender				0.6209
Male	98	60	38	
Female	62	35	27	
Smoking				0.1802
No	58	30	28	
Yes	102	65	37	
Tumor size (cm)				0.0023^∗∗^
≤3	85	60	25	
>3	75	35	40	
TNM stage				0.0157^∗^
I/II	90	61	29	
III/IV	70	34	36	
Depth of invasion				0.0037^∗∗^
T1/T2	82	58	24	
T3/T4	78	37	41	
Lymph node metastasis				0.0135^∗^
N0	112	74	38	
N1	48	21	27	
Distant metastasis				<0.0001^∗∗∗^
M0	107	76	31	
M1	53	19	34	

^∗^ *p* < 0.05, ^∗∗^*p* < 0.01, ^∗∗∗^*p* < 0.001.

**Table 2:** Primer sequences used for qRT-PCR.

ID	Sequence (5′-3′)
*β*-Actin F	GGGCATCCTGACCCTCAAG
*β*-Actin R	TCCATGTCGTCCCAGTTGGT
U6 snRNA F	CTCGCTTCGGCAGCACA
U6 snRNA R	AACGCTTCACGAATTTGCGT
hsa_circ_0000518 F	AGGTGAGTTCCCAGAGAACGG
hsa_circ_0000518 R	AGTGGAGTGACAGGACGCA
RPPH1 F	GTCACTCCACTCCCATGTCC
RPPH1 R	CAGCCATTGAACTCACTTCG
miR-326 F	CCTCTGGGCCCTTCCTCCAG
miR-326 R	GCTGTCAACGATACGCTACCTA
miR-330-3p F	ACACTCCAGCTGGGGCAAAGCACACGGCCTG
miR-330-3p R	TGGTGTCGTGGAGTCG
miR-1296 F	TTGTTAGGGCCCTGGCTC
miR-1296 R	CAGTGCAGGGTCCGAGGTAT
SLC1A5 F	CTCCAGCCCTCGGGAGTAAA
SLC1A5 R	CGGATAAGCAGCTCCCCTTC

## Data Availability

The data used to support the findings of this study are included within the article.
